# Reaction Decoder Tool (RDT): extracting features from chemical reactions

**DOI:** 10.1093/bioinformatics/btw096

**Published:** 2016-02-22

**Authors:** Syed Asad Rahman, Gilliean Torrance, Lorenzo Baldacci, Sergio Martínez Cuesta, Franz Fenninger, Nimish Gopal, Saket Choudhary, John W. May, Gemma L. Holliday, Christoph Steinbeck, Janet M. Thornton

**Affiliations:** ^1^European Molecular Biology Laboratory, European Bioinformatics Institute EMBL-EBI, Wellcome Trust Genome Campus, Hinxton, Cambridge CB10 1SD, UK; ^2^Congenica Ltd, Wellcome Trust Genome Campus, Hinxton, Cambridge CB10 1SA, UK; ^3^DISI, University of Bologna, V.Le Risorgimento 2, Bologna, Italy; ^4^Cancer Research UK Cambridge Institute, University of Cambridge, Li Ka Shing Centre, Robinson Way, Cambridge CB2 0RE, UK; ^5^Michael Smith Laboratories, the University of British Columbia, Vancouver, British Columbia V6T 1Z4, Canada,; ^6^Innovation Centre (Unit 23), Cambridge Science Park, NextMove Software Ltd, Cambridge CB4 0EY, UK; ^7^Bioengineering, UCSF School of Pharmacy, San Francisco, CA 94158, USA

## Abstract

**Summary:** Extracting chemical features like Atom–Atom Mapping (AAM), Bond Changes (BCs) and Reaction Centres from biochemical reactions helps us understand the chemical composition of enzymatic reactions. Reaction Decoder is a robust command line tool, which performs this task with high accuracy. It supports standard chemical input/output exchange formats i.e. RXN/SMILES, computes AAM, highlights BCs and creates images of the mapped reaction. This aids in the analysis of metabolic pathways and the ability to perform comparative studies of chemical reactions based on these features.

**Availability and implementation:** This software is implemented in Java, supported on Windows, Linux and Mac OSX, and freely available at https://github.com/asad/ReactionDecoder

**Contact**: asad@ebi.ac.uk or s9asad@gmail.com

## 1 Introduction

Large-scale chemical reaction databases such as KEGG (Kanehisa *et al.*, 2013), BRENDA (Chang *et al.*, 2015), Rhea (Alcántara *et al.*, 2012) and MetaCyc (Latendresse *et al.*, 2012) link reactions to enzymes and provide data-mining opportunities for novel pathways (Hatzimanikatis *et al.*, 2004; Rahman, 2007), and the discovery of drugs, natural products and green chemistry. One of the primary bottlenecks for automated analyses of these chemical reactions comes from the realizations of the imperfect quality of data, such as unmapped or unbalanced reactions. Accurate Atom–Atom Mapping (AAM)—the one-to-one correspondence between the substrate and product atoms (Gasteiger, 2003), will lead to correct prediction of bond changes (BCs) (Rahman *et al.*, 2014) and the ability to locate the fate of interesting atoms or substructure across metabolic networks (Faulon and Bender, 2010) etc. Linking novel pathways or optimizing pathways of biological/commercial relevance demands better understanding of metabolic routes (Rahman, 2007) and pathway annotation (May *et al.*, 2013).

We present Reaction Decoder Tool (RDT), a robust open source software for mining reaction features, i.e. BCs, reaction centres and to calculate similarity between reactions etc. The algorithm and competing tools have been published in our previous article (Rahman *et al.*, 2014). Here we present the source code with relevant changes and below mentioned features.

## 2 Features

**Fig. 1:**
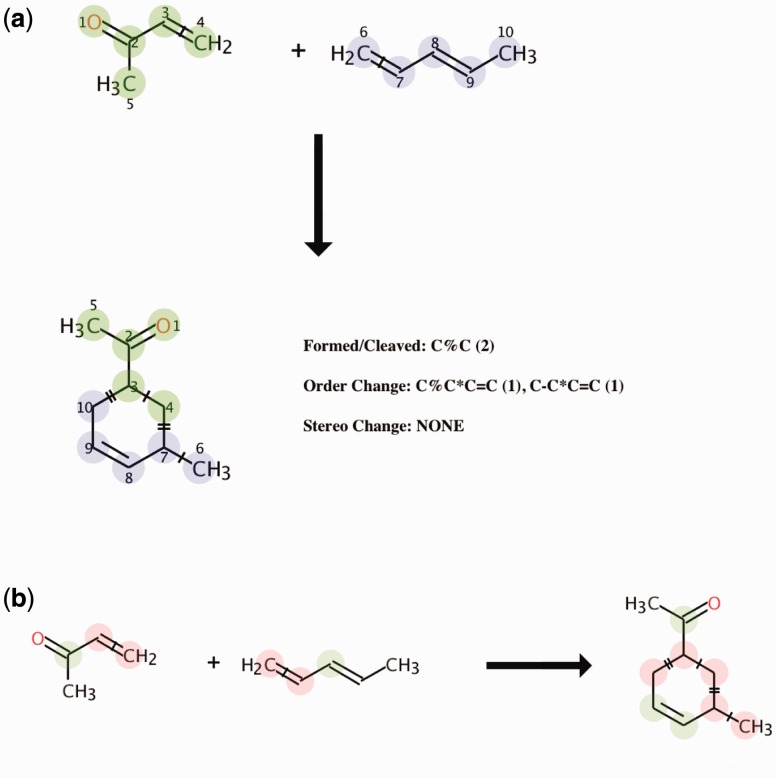
(a) AAM performed by RDT and the resulting BCs (i.e. formed/cleaved: C%C (Ring), Order Change: C%C*C = C, C-C*C = C) and **(b)** reaction centres are highlighted in pink colour in the image, where as neighbouring atoms are highlighted in green

BCs in chemical reactions refers to the cleavage and formation of chemical bonds, changes in bond order and stereo changes, which are due to chemical processes such as chiral inversions or cis-trans isomerization(s). In the chemical reaction diagram and tables atoms are connected by a bond i.e. single ‘–‘, double ‘=’or ring ‘%’ etc. (Fig. 1a), for instance **C–C** means a single carbon–carbon bond that is cleaved (‘|’) or formed in the reaction (‘‖’). Bond order changes are represented by star ‘*’, e.g. C–C * C = C means a single carbon–carbon bond turning into double carbon-carbon bond or vice versa. Stereo changes are represented as atoms that change their absolute configuration, for instance **C(R/S)** means a carbon atom that changes from R to S configuration. A reaction centre is the collection of atoms and bonds that are changed during the reaction (Warr, 2014), also known as the local atomic environment around the atoms involved in BCs.

The key features of this tool are:

- Ability to perform AAM on chemical reactions catalyzed by enzymes (Fig. 1).
- In a balanced reaction, the total number of atoms on the left side of the equation (Reactant), equals the total number of atoms on the right (Product). In unbalanced reactions a best ‘guess’ is made.
- Generates images of the mapped reactions where matching substructures are highlighted.
- Generates reaction patterns and BCs for input reactions.
- The input format and resulting mapped reaction with AAM information can be SMILES or RXN file (Gasteiger, 2003).
- The SMSD (Rahman *et al.*, 2009) and CDK (Steinbeck *et al.*, 2006) are used to process chemical information.

## 3 Usage and applications

*Tool*s like EC-Blast (Rahman *et al.*, 2014), FunTree (Sillitoe and Furnham, 2016), MACiE (Holliday *et al.*, 2012) etc. use RDT in the background to mine and extract chemical information from thousands of enzyme reactions. The success rate of mapping is >99% when compared with manual AAM mappings (Rahman *et al.*, 2014). Originally developed to explore enzyme reactions, the tool is also useful to explore any kind of organic chemical reaction (Martínez Cuesta *et al.*, 2014).

## 4 Conclusion

Reaction Decoder is a robust tool to compute AAM and extract chemical features and calculate similarity between chemical reactions. This is coded in java and optimized to run as a computationally asynchronous process. It is distributed under GNU-GPL V3 license.
